# Solution structure of a 2:1 complex of anticancer drug XR5944 with TFF1 estrogen response element: insights into DNA recognition by a bis-intercalator

**DOI:** 10.1093/nar/gku219

**Published:** 2014-04-07

**Authors:** Clement Lin, Raveendra I. Mathad, Zhenjiang Zhang, Neil Sidell, Danzhou Yang

**Affiliations:** 1 College of Pharmacy, University of Arizona, 1703 E. Mabel Street, Tucson, AZ 85721, USA; 2 Department of Gynecology and Obstetrics, Emory University School of Medicine, Atlanta, GA 30322, USA; 3 Department of Chemistry, University of Arizona, Tucson, AZ 85721, USA; 4 BIO5 Institute, University of Arizona, Tucson, AZ 85721, USA; 5 The Arizona Cancer Center, Tucson, AZ 85724, USA

## Abstract

XR5944, a deoxyribonucleic acid (DNA) bis-intercalator with potent anticancer activity, can bind the estrogen response element (ERE) sequence to inhibit estrogen receptor-α activities. This novel mechanism of action may be useful for overcoming drug resistance to currently available antiestrogen treatments, all of which target the hormone-receptor complex. Here we report the nuclear magnetic resonance solution structure of the 2:1 complex of XR5944 with the naturally occurring TFF1-ERE, which exhibits important and unexpected features. In both drug–DNA complexes, XR5944 binds strongly at one intercalation site but weakly at the second site. The sites of intercalation within a native promoter sequence appear to be context and sequence dependent. The binding of one drug molecule influences the binding site of the second. Our structures underscore the fact that the DNA binding of a bis-intercalator is directional and different from the simple addition of two single intercalation sites. Our study suggests that improved XR5944 bis-intercalators targeting ERE may be designed through optimization of aminoalkyl linker and intercalation moieties at the weak binding sites.

## INTRODUCTION

Breast cancer is the most commonly occurring cancer in women and it remains a leading cause of cancer deaths in women despite major advances in treatment over the past 20 years. Estrogens are steroid hormones that play significant roles in the genesis, development and metastasis of breast cancer (1). Estrogen (E2) responses in breast cancer cells are predominantly mediated by the estrogen receptor-α (ERα), a ligand-activated transcription factor (2). ERα regulates transcription of target genes through direct binding to its cognate recognition sites, known as estrogen response elements (EREs), or by modulating the activity of other deoxyribonucleic acid (DNA) binding transcription factors at alternative DNA sequences (3). ERα modulation by endocrine therapy is the primary means to treat ERα-positive breast tumors (4). These antiestrogen treatments are comprised of selective ER modulators (e.g. tamoxifen), which impair the hormone-receptor complex (5), and aromatase inhibitors (AIs, e.g. anastrazole), which inhibit E2 production (6–7). Unfortunately, a significant fraction (∼20–50%) of ERα-positive breast tumors fail to respond (8) or eventually develop resistance to antiestrogen treatments (9). Hence, there remains an urgent need for new and effective agents that overcome the resistance to existing endocrine therapies.

**Figure 1.**
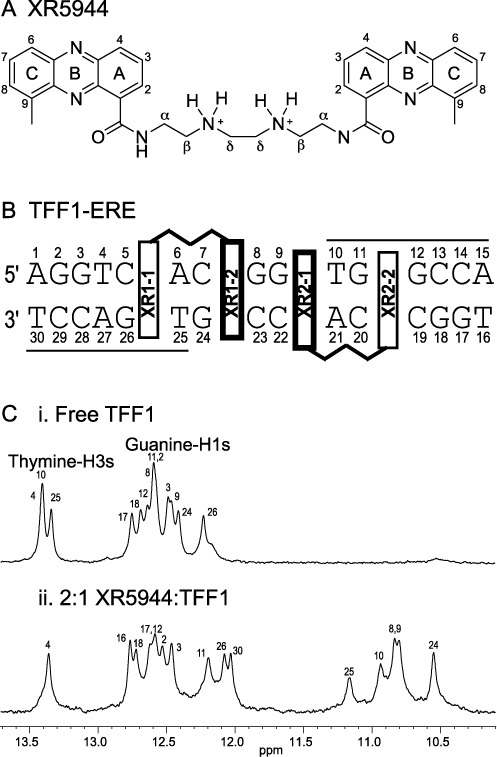
(**A**) Chemical structure of XR5944 with the atom numbering system. (**B**) The TFF1 ERE DNA sequence used in this paper with numbering. The binding sites of the two XR5944 molecules are shown schematically, with the strong binding sites of the XR1–2 phenazine and the XR2–1 phenazine shown in darker boxes, and the weak binding sites of XR1–1 and XR2–2 shown in lighter boxes. The ERE half-sites are marked by solid lines. (**C**) The imino regions of 1D ^1^H NMR spectra of the free TFF1-ERE DNA duplex (i) and the 2:1 XR5944:TFF1 complex (ii) with proton assignments. Conditions: 25°C, pH7, 50-mM sodium phosphate.

We previously showed that XR5944, a DNA bis-intercalator with potent anticancer activity, is capable of inhibiting ERα-mediated transcriptional responses via its ability to block the binding of ERα to the ERE sequence (10). This novel mechanism of action has the potential to overcome drug resistance of currently available antiestrogen treatments, which all target the hormone-receptor complex and are susceptible to drug resistance due to mutations in ERα, or post-translational modifications which render it constitutively active in the absence of ligand (11). XR5944 is highly potent against human tumor cell lines and xenograft models including breast cancers (12–13). This compound (Figure 1A) was originally developed as a dual topoisomerase I/II inhibitor (14), but was later found to be a transcription inhibitor instead (15–16). Using artificially designed palindromic sequences, we showed that the preferred bis-intercalating sequence of XR5944 is the palindromic 5′-TGCA in which XR5944 bis-intercalates at the two (TpG):(CpA) sites sandwiching two central G:C base pairs between the two intercalating phenazine rings, with its carboxamide aminoalkyl linker lying in the DNA major groove (17). The consensus ERE is an inverted repeat comprised of two ERE half-sites separated by three bases: 5′-AGGT**CA**nnn**TG**ACCT where nnn is known as the tri-nucleotide spacer (18–20). The 5′-(CpA) or the 5′-(TpG) site is found in each half-site of the consensus ERE, thus the blocking activity of XR5944 was predicted to have a certain degree of specificity for interfering with the binding of ERα to the ERE sequences. This prediction was supported by the data showing that XR5944 neither inhibited transactivation of the Sp1 consensus binding site 5′-GGGGCGGGGC (10) nor blocked the binding of transcription factor NF-κB to its consensus promoter sequence 5′-GGGACTTTCC (17), with both sequences lacking 5′-TG motifs.

However, the preferred bis-intercalating sequence 5′-T|GC|A (| denotes the drug intercalation site) for XR5944 is not present in the ERE sequence, thus the binding characteristics of XR5944 to ERE sequences could be different. Indeed, recently we have shown that the spacer sequence affects the binding affinity and specificity of XR5944 with ERE sequences, which consequently affects the efficacy of XR5944 inhibition of ERα-mediated transcriptional responses at consensus EREs (21). Further, we found that XR5944 binds the ERE in the promoter of the estrogen-responsive target gene trefoil factor 1 (*TFF1*, previously designated *PS2*) (Figure 1B) with high affinity (21). This makes TFF1-ERE a promising candidate for structural characterization of the XR5944 binding with ERE. Here, we report the nuclear magnetic resonance (NMR) solution structure of the 2:1 complex of XR5944 with TFF1-ERE DNA. To our knowledge, this is the first bis-intercalator complex structure with a naturally occurring promoter sequence. Interestingly, the binding sites of XR5944 are different from previously predicted and show some highly unexpected features. Our results show that, in each drug–DNA complex, XR5944 binds strongly at one intercalation site but weakly at the second intercalation site. In addition, our results explain why the spacer sequences of ERE can affect the XR5944-DNA binding. Understanding the precise binding mode of XR5944 to a naturally occurring ERE sequence would provide an important basis for the design and development of DNA bis-intercalators specifically targeting ER–ERE interactions for new breast cancer therapeutics.

## MATERIALS AND METHODS

### Sample preparation

DNA oligonucleotides of sense and complementary TFF1-ERE sequences, 5′-AGGTCACGGTGGCCA and 5′-TGGCCACCGTGACCT, were synthesized at 1-μmol scale using β-cyanoethylphosphoramidite solid-phase chemistry on an Expedite 8809 nucleic acid synthesis system (Applied Biosystems, Inc.) with dimethoxytrityl (DMT)-ON setting and were purified using C18 reverse-phase high pressure (or high performance) liquid chromatography or MicroPure II Columns from BioSearch Technologies (Novato, CA, USA), as described previously (17,22–23). XR5944 was provided by Xenova Ltd (Slough, UK). DNA concentrations were determined by ultraviolet absorbance at 260 nm (extinction coefficients are 147 400, 140 500 and 242 632.5 l mol^−1^ cm^−1^ for the TFF1 sense strand, complement strand and duplex, respectively). The NMR samples were prepared by dissolving DNA oligonucleotide powder into 50-mM sodium phosphate buffer at pH 7 in either pure D_2_O (98%) or D_2_O/H_2_O (10%/90%). The D_2_O samples were lyophilized and resuspended in 99.98% D_2_O two more times. The TFF1 duplex sample was prepared by titrating one strand into the solution of the complimentary strand. The titration was monitored through the DNA imino signals in 1D ^1^H NMR spectra. The DNA–drug complexes were prepared by adding an appropriate amount of drug stock solution to the DNA sample, followed by lyophilization and re-dissolution in D_2_O. The final concentrations of DNA oligonucleotides were 1–2 mM.

### NMR experiments

Both 1D and 2D NMR experiments were carried out on a Bruker Avance 600-MHz spectrometer as described earlier (17,22–25). The imino protons and base H8/H6 protons of guanine/thymine can be unambiguously assigned by 1D ^15^N-edited GE-JRSE HMQC experiments (26). For this purpose, we prepared DNA samples site-specifically labeled at each guanine and thymine of TFF1-ERE with low-enrichment (6%) incorporation of ^15^N-labeled guanine or thymine, respectively (27). Standard homonuclear 2D NMR experiments were used to assign the non-exchangeable proton chemical shifts of the free TFF1-ERE DNA and DNA–XR5944 complex, including double-quantum filtered correlation spectroscopy (DQF-COSY), total correlation spectroscopy (TOCSY) and nuclear Overhauser effect spectroscopy (NOESY), at temperatures of 5, 15, and 25°C. The mixing times were set from 50–250 ms for NOESY, and at 30 ms and 60 ms for TOCSY. The NMR experiments for samples in water solution were performed with WATERGATE or jump-return (NOE11) water suppression techniques. The relaxation delay was set to 2 s. The acquisition data points were set to 4096 × 512. The 60° shifted sine bell functions were applied to both dimensions of NOESY and TOCSY spectra. The five-order polynomial functions were employed for the baseline corrections. The final data points were 4096 × 1024. Peak assignment and integration were achieved using Sparky (UCSF). Distances between non-exchangeable protons were assigned based on the nuclear overhauser effect (NOE) crosspeaks integrated at 50–250-ms mixing times. The peak volumes were referenced using the distance H5-H6 of cytosine (2.45 Å) with limits on distance restraints set to 20% variance. When appropriate, spin diffusion adjustments were made based on variable mixing time experiments. Unsolved protons were replaced by pseudo-atoms and the appropriate correction was applied to the measured distance.

### Distance restrained molecular dynamics simulation

Structure calculations were performed using NOE-restrained molecular dynamics (RMD) simulation in the program XPLOR (version 3.851) (28). The starting model of the 2:1 XR5944–TFF1 complex was constructed in Insight II 2000.1 (Accelrys, CA, USA), with the intercalation site conformations deduced from the NOE data. The partial charges were obtained from X-PLOR or from the representative fragments in Insight II. The CHARMM force field was used for the calculation. The skewed bi-harmonic energy function was used for distance constraints from NOE data. A total of 669 distances were used in the NOE-restrained dynamics calculations. A distance-dependent dielectric constant was used in the calculations to simulate the aqueous environment.

The system was first equilibrated at 300 K for 20 ps, with the force constants of 1 kcal/mol·A2 for all restraints. The resulting structures were then equilibrated at 1000 K for 3 ps. The force constants were gradually scaled to the final values of 30 and 60 kcal/mol·A2 for NOE and hydrogen bond restraints, respectively, during the subsequent 24-ps simulation. Subsequent RMD and cooling simulation was carried out at temperature reduced by 25 K with 1000 time steps of 3 fs each cycle until the final temperature reached 300 K. The system was then equilibrated for an additional 3 ps. The coordinates saved during the last 3.0 ps were averaged. The resulting structures were further subjected to 250 steps of energy minimization. The 10 best molecules were selected based on their minimal energy terms and minimal number of NOE violations.

Aqueous phase molecular dynamics calculations were performed in Insight II using periodic boundary conditions. The system first underwent energy minimization at 300 K for 1000 steps. Distance restraints were then applied for an additional 1000 steps of energy minimization. Molecular dynamics simulations were then run for 5 ps at 300 K. This was repeated to generate a separate ensemble of structures, which were then compared with XPLOR results to confirm the structure features.

### Fluorescent intercalator displacement (FID) assay

Binding of XR5944 to TFF1-ERE can displace intercalated ethidium bromide (EtBr) and quench the fluorescence from the EtBr–DNA complex, thus allow the measurement of binding fraction. DNA (20 μM, 5-mM sodium phosphate buffer, pH 7) was incubated with EtBr (1 mM, deionized (DI) water) for 1 h. Samples were then transferred to a 96-well plate; XR5944 (5-mM stock in DI water) and water was added into the EtBr–DNA solution to get a final DNA concentration of 16.7 μM and final XR5944 concentrations between 0 and 100 μM in 120-μl volume per well. Three samples were prepared per XR5944 concentration. The fluorescence intensities were obtained using a microplate reader (Molecular Devices Gemini XS) at 25ºC. Samples were excited at 510 nm, and the fluorescence was measured at 590 nm six times per sample. Fluorescence readings were corrected for baseline fluorescence of free EtBr and the fluorescence of XR5944 in complex with DNA.

## RESULTS

### XR5944 binds TFF1-ERE DNA duplex at a 2:1 ratio

It has been shown previously that the preferred binding sequence of XR5944 (Figure 1A) is 5′-(T|GC|A), with the two phenazine chromophores bis-intercalating at the two (TpG):(CpA) sites, sandwiching two G:C base pairs (17). The 15-mer TFF1-ERE sequence 5′-(AGGTCACGGTGGCCA):(TGGCCACCGTGACCT)-3′ (Figure 1B) contains one 5′-CpA and one 5′-TpG site. Each Watson–Crick base pair contains one imino proton, i.e. guanine H1 for G:C/C:G base pairs and thymine H3 for T:A/A:T base pairs. Imino protons of TFF1-ERE DNA were detectable in 1D ^1^H NMR for all non-terminal base pairs, with three thymine H3 protons for three non-terminal T:A/A:T base pairs (13.2–13.6 ppm) and 10 guanine H1 protons for 10 G:C/C:G base pairs (12–12.8 ppm) (Figure 1C). The imino protons of the two terminal A:T base pairs are not detectable because of their rapid exchange with water due to the end-fraying effect (17,29). Upon addition of XR5944, a new set of imino proton peaks from the drug-bound DNA started to emerge, whereas imino proton peaks from the free DNA started to vanish (Supplementary Figure S1A). The observation of two sets of imino peaks, one from the free DNA and another from the complex DNA, indicates that XR5944 binds the TFF1-ERE at a medium-to-slow exchange rate on the NMR time scale. The upfield shifting of imino protons of the bound DNA (Figure 1C-ii) is characteristic of an intercalating drug binding mode (17,30). The binding stoichiometry of XR5944 with the TFF1-ERE DNA appeared to be 2:1, as no further spectral change was observed at higher drug equivalence (Supplementary Figure S1A). At the drug equivalence of 2, the imino proton peaks from the free DNA almost completely vanished, leaving the new set of imino proton peaks from the drug–DNA complex (Figure 1C-ii). The imino protons from the two terminal A:T base pairs, T_30_ and T_16_, respectively, are observed in the XR5944–DNA complex (Figure 1C-ii), indicating that the binding of XR5944 reduced the end-fraying effect by stabilizing the TFF1-ERE DNA duplex.

### Base proton assignment using site-specific ^15^N-labeling

**Figure 2.**
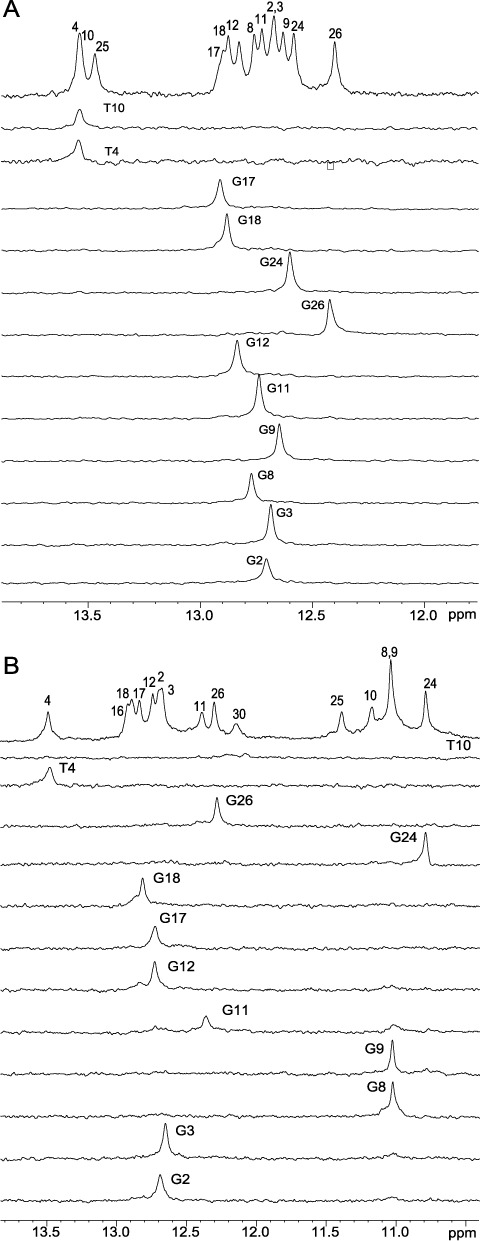
The imino proton assignments obtained using 1D ^15^N-edited HMQC experiments on site-specific-labeled TFF1 DNA for (**A**) free TFF1-ERE DNA and (**B**) 2:1 XR5944:TFF1 complex.

We prepared DNA samples labeled site-specifically at each guanine and thymine, respectively, of TFF1-ERE with low-enrichment (6%) incorporation of ^15^N-labeled bases. The guanine base H1 and H8 protons and the thymine base H3 and H6 protons can be detected by 1D ^15^N-edited HMQC experiments (26–27) (Figure 2A and Supplementary Figure S2A). As each base pair contains one imino proton, it can be unambiguously assigned using site-specifically labeled DNA (Figure 1C-i and Supplementary Table S1). We then prepared 2:1 XR5944–TFF1 complexes with each labeled DNA and detected guanine H1 imino protons and T4H3, as well as guanine H8 and T4H6 protons by ^15^N-edited HMQC (Figure 2B, Supplementary Figure S2B and Supplementary Table S1). The missing proton assignments of several thymines were obtained using a sequential assignment method (see below).

### Proton assignment of the free TFF1 DNA and the 2:1 XR5944–TFF1 complex

**Figure 3.**
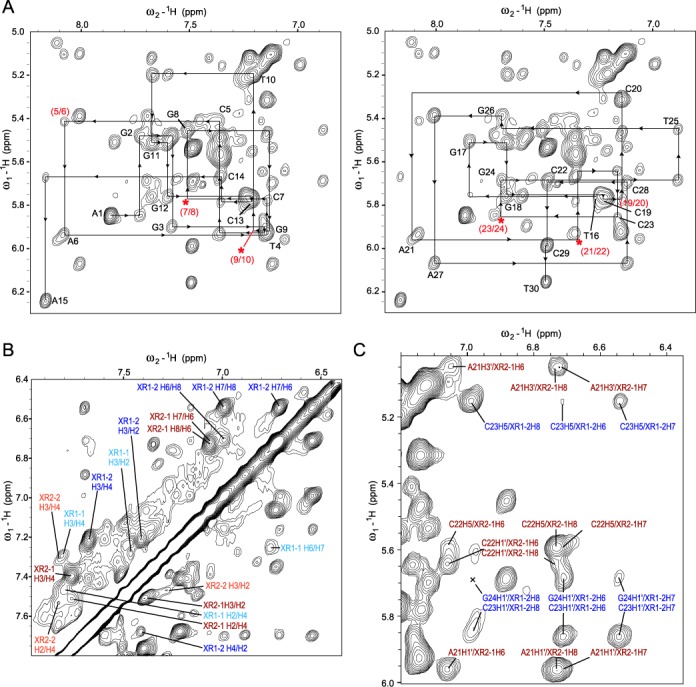
(**A**) The expanded aromatic-H1′ region of the 2D-NOESY spectrum of the 2:1 XR5944:TFF1 complex. The sequential assignment pathways are shown for the DNA sense strand (A_1_–A_15_) (left) and complement strand (T_16_–T_30_) (right). The intra-residue TFF1 DNA H8/H6-H1′ NOEs are labeled with residue names. The missing (asterisks) or weak connectivities are labeled in red. The expanded aromatic–aromatic (**B**) and H1′/H5-aromatic region (**C**) of the 2D-NOESY spectrum of the 2:1 XR5944:TFF1 complex. Assignments are shown for the intra-XR5944 NOEs (B) and for the intermolecular drug–DNA NOEs (C). NOEs involving XR-1 and XR-2 phenazines are in blue and red respectively.

We collected complete sets of 2D-NOESY, TOCSY and COSY NMR data in both water and D_2_O for both the free TFF1 DNA duplex (Supplementary Figure S3) and 2:1 XR5944–TFF1 complex (Figure 3). Starting from the assignments of the imino protons and G-H8/T-H6 protons obtained from ^15^N-labeled experiments (Figure 2 and Supplementary Figure S2), complete proton assignments of the free TFF1 DNA duplex and the 2:1 XR5944–TFF1 complex were achieved using a sequential assignment method (Figure 3A, Supplementary Figure S3 and Supplementary Table S1). Free 15-mer TFF1 DNA forms a regular B-type double helix in solution, as indicated by standard sequential connectivities (Supplementary Figure S3) and intra-sugar interactions in NOESY and COSY spectra. The spectral linewidth of the 2:1 XR5944–TFF1 complex is in general broader than that of the free TFF1 DNA (Figure 1C), suggesting a higher degree of internal motion of the drug–DNA complex. The glycosidic torsion angles of all nucleotides in the 2:1 XR5944–TFF1 complex are in the anti configuration, as indicated by the intraresidue H6/H8-H1′ NOE intensities (Figure 3A). However, the sequential NOE connectivities of the (*n*) aromatic H6/H8 protons to the (*n* + 1) H1′/H2′/H2″ protons typical for double-helical B-DNA are interrupted at several steps. Specifically, the connectivities at C_7_pG_8_:C_23_pG_24_ and G_9_pT_10_:A_21_pC_22_ steps are missing, while the connectivities at C_5_pA_6_:T_25_pG_26_ and G_11_pG_12_:C_19_pC_20_ steps are very weak (Figure 3A). This indicates that the two bis-intercalation binding sites of XR5944 are C_5_|pA_6_C_7_|pG_8_:C_23_|pG_24_T_25_|pG_26_ and G_9_|pT_10_G_11_|pG_12_:C_19_|pC_20_A_21_|pC_22_ (Figure 1B), as the intercalation of XR5944 at these positions breaks the normal base-stacking interactions by pushing the two adjacent base pairs apart.

The protons of the two XR5944 molecules were assigned using NOESY in combination with COSY and TOCSY (Supplementary Table S1). The drug phenazine ring proton H8 (Figure 1A) was assigned by a strong NOE crosspeak with the drug C9 methyl group. This led to the assignment of its vicinal proton H7, and then of H6. The drug H3 proton was identified by having two COSY crosspeaks with both H2 and H4, while H2 was confirmed by a number of NOE crosspeaks with its flanking DNA bases.

### Each XR5944 has one strong intercalation site and one weak site within TFF1-ERE DNA

**Figure 4.**
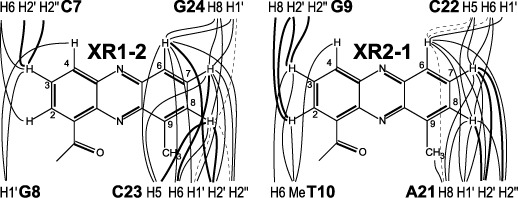
Schematic diagram of intermolecular NOEs between TFF1 DNA protons and the two strong intercalating drug moieties: XR1–2 of the first XR5944 molecule and XR2–1 of the second XR5944 molecule. The bold solid lines, solid lines and dashed lines correspond to strong, medium and weak NOE interactions, respectively.

Interestingly, while protons of the bound DNA are well defined and can be unambiguously assigned, only one intercalating phenazine ring of each XR5944 molecule, i.e. XR1–2 and XR2–1 (Figure 1B), is well defined with all proton resonances being unambiguously assigned (Figure 3B). Protons of the second intercalating phenazine rings of the two XR5944 molecules, i.e. XR1–1 and XR2–2, are much broader and less well defined (Figure 3B). These data indicate that, for each XR5944 molecule, only one phenazine moiety binds strongly at the intercalating site, whereas the second phenazine binds weakly and is more dynamic at the intercalating site. This result was supported by the intermolecular NOE crosspeaks between XR5944 and TFF1-ERE DNA. Clearly defined intermolecular NOE crosspeaks were only observed for the XR1–2 and XR2–1 strong binding sites of the first and second XR5944 molecules, respectively (Figures 3C and 4). These intermolecular drug–DNA NOEs (Figure 4) defined the binding sites and modes of XR5944.

For the first strong binding site XR1–2 (Figure 1B), the ring C aromatic protons, i.e. H6, H7 and H8, showed clear NOEs with DNA base and sugar protons of C_23_ and G_24_, while the ring A aromatic protons, i.e. H2, H3 and H4, showed NOEs with DNA C_7_ and G_8_ protons (Figure 4), indicating that XR1–2 intercalates at the (C_7_pG_8_):(C_23_pG_24_) step. In particular, C_23_H5 (major groove side) showed a very strong NOE crosspeak with H8 of XR1–2, a medium NOE with H7 and a weak NOE with H6. In contrast, C_23_H1′ (minor groove side) showed a weak NOE with H8 of XR1–2, a strong NOE with H7 and a medium strong NOE with H6 (Figures 3C and 4). XR1–2 H6 also showed a clear NOE with G_24_H1′ (minor groove side) (Figure 4). These NOE interactions placed the carboxyamide linker, which is at the same side of ring C H8, in the major groove of the TFF1 DNA duplex. Consistently, H4 (and H3) of ring A showed clear NOEs with G_8_H1′ in the minor groove of DNA, whereas H2 of ring A, located at the same side of the carboxyamide linker, showed clear NOE with C_7_H6 in the DNA major groove (Figure 4). Thus the intermolecular NOEs suggested a parallel base-stacking intercalation of XR1–2, with the long axis of its phenazine parallel to the long axes of the flanking DNA base pairs and its aminoalkyl linker in the major groove of the DNA duplex.

For the second strong binding site XR2–1 (Figure 1B), intermolecular NOEs suggested a parallel base-stacking intercalation at the (G_9_pT_10_):(A_21_pC_22_) step, with the linker in the major groove of the DNA duplex. For example, H6 of ring C showed strong NOEs with both the C_22_H1′ and A_21_H1′ (minor groove) (Figures 3C and 4), while H8/H7 of ring C showed strong NOEs with C_22_H5 and A_21_H8 (major groove) (Figures 3C and 4); H2 of ring A showed a strong NOE with G9H8 (major groove) (Figure 4).

**Figure 5.**
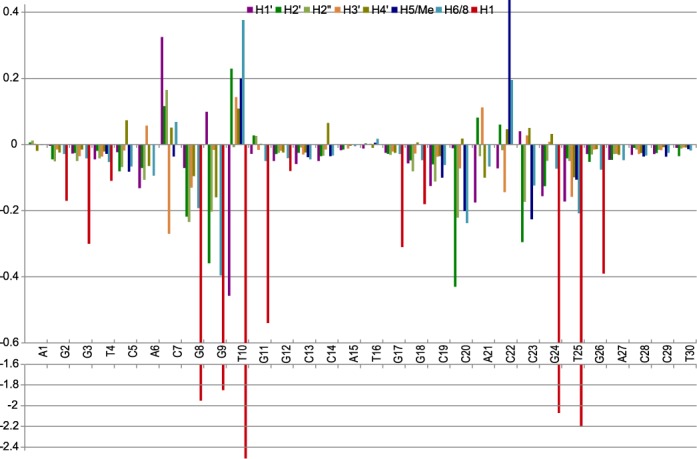
The chemical shift difference of DNA protons between the free TFF1-ERE DNA duplex and the 2:1 XR5944:TFF1 complex at 25°C. The corresponding residues are shown in Figure 1B.

The bis-intercalation of XR5944 at the C_5_|pA_6_C_7_|pG_8_:C_23_|pG_24_T_25_|pG_26_ and G_9_|pT_10_G_11_|pG_12_:C_19_|pC_20_A_21_|pC_22_ sites of TFF1-ERE sequence (Figure 1B) was also supported by NMR titration data (Figure 1C) and proton chemical shift changes between the free and bound DNA (Figure 5). In the XR5944–TFF1 complex, the imino protons of base pairs A_6_:T_25_, C_7_:G_24_, G_8_:C_23_, G_9_:C_22_ and T_10_:A_21_ were the most affected by drug binding (Figure 1C-ii) and showed large upfield shifts (2.2, 2.07, 1.95, 1.85 and 2.51 ppm, respectively; Figure 5). Such upfield shifts, induced by the ring-current effects of the intercalating XR5944 phenazine, are characteristic of an intercalative mode of drug binding. The large chemical shift changes were also observed for other protons at the binding sites (Figure 5). However, chemical shift change patterns appeared to be different at different binding sites, suggestive of diverse binding positions of phenazine rings. The chemical shift changes at C_5_|pA_6_:T_25_|pG_26_ were smaller as compared to other binding sites (Figure 5), while the G_11_ imino proton was not shifted much by drug binding (Figure 1C), consistent with the weak binding sites of XR2–2 and XR1–1.

### NMR structure calculation

**Figure 6.**
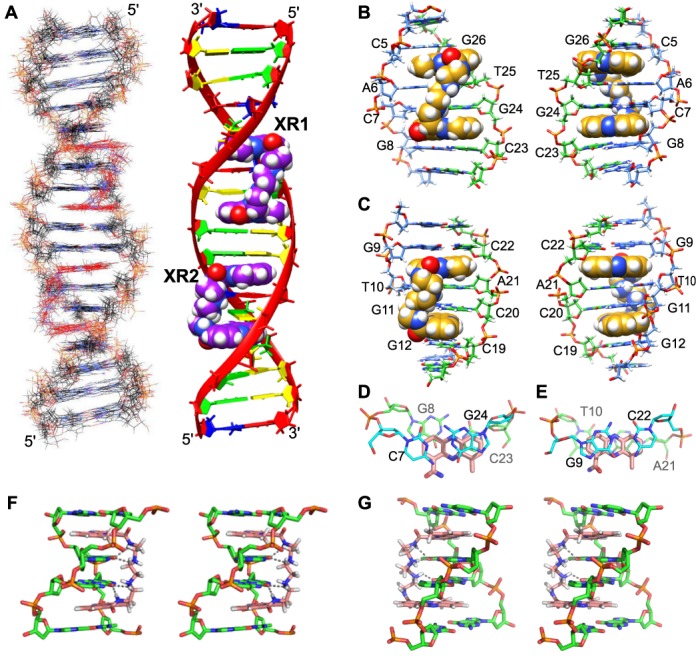
(**A**, Left) Superimposed 10 final structures of the 2:1 XR5944:TFF1 complex by NOE-RMD structure calculation (PDB ID 2mg8). (Right) A representative model of the NOE-refined 2:1 XR5944:TFF1 complex structure. The XR5944 molecules are shown in CPK model. Adenine, thymine, guanine and cytosine are red, blue, green and yellow, respectively. (**B**)–(**G**) The binding interactions within the two XR5944 complexes. (B) and (C) The binding of the first XR5944 molecule XR1 (B) and the second XR5944 molecule XR2 (C) in the 2:1 XR5944:TFF1 complex, viewed from the major groove (left) and the minor groove (right). The XR5944 molecules are shown in CPK. DNA strands are colored by atom, with carbon atoms of one strand in green and another strand in light blue. (D) and (E) Base-stacking interactions of XR1–2 (D) and XR2–1 (E) intercalation site. Carbon atoms of XR5944 are colored salmon, whereas carbon atoms of DNA are colored by base pair. Nitrogen atoms are blue, oxygen atoms are red, and phosphorus atoms are orange. (F) and (**G**) H-bonding interactions (black dashed lines) between the DNA major groove and the carboxamide aminoalkyl linker of XR1 (F) and XR2 (G) in stereo view. Carbon atoms of XR5944 are colored salmon and hydrogen atoms are white. DNA strands are colored by atom, with carbon atoms in green.

A starting model of the 2:1 XR5944–TFF1 complex was constructed using the above-mentioned information in INSIGHT-II. The intermolecular drug–DNA NOEs clearly defined that the aminoalkyl linkers of the two XR5944 molecules were positioned in the major groove of the DNA duplex. For the two strong binding sites, i.e. XR1–2 and XR2–1, the orientation and stacking position of the XR5944 phenazine rings were well defined by drug–DNA intermolecular NOEs (Figures 3C and 4) and used for model construction. For the two weak binding sites, i.e. XR1–1 and XR2–2, however, drug–DNA intermolecular NOEs were not well defined, thus the drug intercalation sites were constructed based on the observed chemical shift changes (Figure 5). The starting model of the XR5944–TFF1 complex was subjected to NOE-RMD and simulated annealing calculations in X-PLOR (28). A total of 669 distance restraints, of which 56 are intermolecular drug–DNA NOEs, were incorporated into the RMD calculation to create a family of NMR-refined structures (Figure 6A, left; PDB ID 2mg8). The statistics of the structures are summarized in Table 1. While no intermolecular NOEs were used for the two weak binding sites, the DNA conformations at the binding sites were well defined based on clear intra-DNA NOEs; the drug conformations at the two weak binding sites are mainly determined by molecular dynamics and energy minimization calculations. As shown in Figure 6A left, the orientations and intercalation positions of the two strong binding sites were converged and well defined, while a much higher conformational flexibility was shown for the XR5944 phenazine rings at the two weak binding sites.

**Table 1.** Structural statistics for 10 refined structures^a^

Number of distance restraints	669
DNA	
Intraresidue	357
Sequential	153
Hydrogen bonds	24
Drug	79
DNA–drug	56
Structure statistics	
NOE violations	
Number > 0.2 Å	2.38 ± 0.99
RMSD of violations (Å)	0.024 ± 0.002
Deviations from ideal geometry	
Bond length (Å)	0.007 ± 0.0007
Bond angle (°)	1.83 ± 0.25
Impropers (°)	0.82 ± 0.01
Pairwise RMSD of heavy atoms (Å)	0.78 ± 0.16

^a^Structures are selected based on least number of restraint violations and lowest energy.

### Asymmetric bis-intercalation interactions of XR5944 molecules with TFF1-ERE DNA

A representative model of NMR-refined complex structure of the XR5944–TFF1 complex is shown in Figure 6A, right. The bis-intercalation binding of the two XR5944 molecules is shown in more detail in Figure 6B and C. The first XR5944 molecule XR1 bis-intercalates at the C_5_|pA_6_C_7_|pG_8_:C_23_|pG_24_T_25_|pG_26_ site (Figure 6B), with the C_7_|pG_8_:C_23_|pG_24_ being the strong binding site for XR1–2, and the second XR5944 molecule XR2 bis-intercalates at the G_9_|pT_10_G_11_|pG_12_:C_19_|pC_20_A_21_|pC_22_ site (Figure 6C), with the G_9_|pT_10_:A_21_|pC_22_ being the strong binding site for XR2–1. The strong binding sites of the two XR5944 drugs are adjacent to each other, so that the XR1–2 and XR2–1 phenazine chromophores are only separated by two base pairs G_8_:C_23_ and G_9_:C_22_, within the CGG spacer in the TFF1-ERE sequence (Figure 1B).

At the strong binding sites, i.e. XR1–2 and XR2–1 of the two XR5944 molecules, both bind in a parallel intercalation mode, with the phenazine rings well stacked with the flanking base pairs (Figure 6D and E). For the XR1–2 intercalation site, C_7_|pG_8_:C_23_|pG_24_, the drug phenazine ring is closer to the sugar backbone of C_23_pG_24_ than that of C_7_pG_8_ and intercalates more within the C_23_pG_24_ step (Figure 6D). H6/H7/H8 of ring C showed clearly stronger NOE interactions with DNA C_23_/G_24_ residues than those between H2/H3/H4 of ring A and C_7_/G_8_ residues (Figure 3C, 4). The base H5/H6 protons of C_23_ are more clearly upfield shifted as compared to those of C_7_ (Figure 5), consistent with the more extensive stacking of the drug phenazine with the C_23_ base (Figure 6D). This asymmetric stacking interaction was not observed for the second strong binding site, i.e. XR2–1 at G_9_|pT_10_:A_21_|pC_22_ (Figure 6E). The intercalation of the XR2–1 phenazine is more symmetric, as reflected in the more evenly distributed NOE interactions of the XR2–1 phenazine ring protons with the A_21_pC_22_ and G_9_pT_10_ steps (Figure 4). The base protons H6/H5/Me of C_22_ or T_10_, respectively, were similarly downfield shifted (Figure 5), indicating their positions outside the stacking drug phenazine ring. Indeed, the phenazine ring appears to better stack with the 6-member rings of the two purine residues at the XR2–1 intercalation site, i.e. A_21_ or G_9_ (Figure 6E).

The intercalation conformations of the two weak binding sites, i.e. XR1–1 and XR2–2, are less well defined and exhibit higher conformational flexibility (Figure 6A). The intercalation site XR1–1 of the first XR5944 molecule appears to be better defined than that of XR2–2 of the second drug, as shown by the more extensive upfield shifting of the imino proton of the A_6_:T_25_ base pair than that of the G_11_:C_20_ (Figure 5). XR1–1 appears to intercalate more evenly within the (C_5_pA_6_):(T_25_pG_26_) step, giving rise to upfield shifting to the base protons of T_25_ (H6/Me), A_6_ (H8), C_5_ (H6/H5) and G_26_ (H8) (Figure 5). In contrast, XR2–2 appears to intercalate more extensively in between the C_19_pC_20_ step versus its base-paired G_11_pG_12_, as shown by larger chemical shift changes observed for the C_19_pC_20_ step, as well as the much smaller upfield shifting observed for the G_11_H1 imino proton (Figures 1C and 5).

For the two strong intercalation sites, the rise between the two intercalated base pairs is ∼6.01 Å at the XR1–2 intercalation site (C_7_|pG_8_):(C_23_|pG_24_) (Figure 6F), and is ∼6.34 Å at the XR2–1 intercalation site G_9_|pT_10_:A_21_|pC_22_ (Figure 6G). The rise for the two weak intercalation sites, i.e. (C_5_|pA_6_):(T_25_|pG_26_) and (G_11_|pG_12_):(C_19_|pC_20_), is 5.95 Å and 5.86 Å, respectively (Figure 6F and G). All four intercalation sites have a rise larger than that of the perpendicular intercalation binding site, as exemplified by anthracycline drugs (30–31), but smaller than the minor-groove binding bis-intercalators such as echinomycin (7.25 Å and 6.81 Å) (32) and triostin A (7.25 Å and 6.99 Å) (33) The DNA double helix is unwound at both XR5944–DNA complexes, with the overall extent of unwinding being 32° for the first drug (XR1) 3-step binding site of C_5_A_6_C_7_G_8_, and 28° for the second drug (XR2) binding site of G_9_T_10_G_11_G_12_ (Figure 6A), as compared to regular B-DNA with an average helical twist of 36° per step. The DNA unwinding of about 4–5° extends to the ±1 steps adjacent to the intercalation site. The unwinding at the G_8_pG_9_:C_22_pC_23_ step between the two XR5944 complexes is larger, i.e. ∼7°, likely due to the combined influence of the two adjacent drug binding sites. In comparison, the unwinding observed for triostin A was more significant, with an average twist of 20° at the intercalation site and 15° at the spanned step (33).

### Major-groove interactions of the two linkers of XR5944 with TFF1-ERE DNA duplex

The carboxamide aminoalkyl linkers of both XR5944 drugs are positioned in the major groove of the TFF1-ERE DNA duplex. The two linker γ-amino groups of XR5944 are protonated and positively charged at pH 7 (Figure 1B), which interact favorably with the electronegative DNA major groove. Specifically, the H-bond acceptors thymine O4 and guanine O6 in the DNA major groove can readily form H-bonding interactions with the linker γ-amino γ-NH_2_(+) and amide NH groups. For the first XR5944 molecule XR1, potential H-bonds appeared to form between T_25_O4 and G_24_O6, and the amide and γ-amino groups of XR1–1 and XR1–2, respectively (Figure 6F). For the second XR5944 molecule XR2, potential H-bonds appeared to form between T_10_O4 and G_11_O6, and the amide and γ-amino groups of XR2–1 and XR2–2, respectively (Figure 6G). Interestingly, the carboxamide aminoalkyl linkers of the two XR5944 molecules in the TFF1–ERE DNA complexes adopt different conformations. The linker of XR1 drug runs diagonally across the major groove with a right-handed twist, such that the drug has the appearance of a Z when viewed from the major groove (Figure 6B). In contrast, the linker of XR2 drug runs across the major groove more vertically (Figure 6C) (see the Discussion section below).

### Binding affinity of XR5944 to TFF1 DNA determined by FID assay

**Figure 7.**
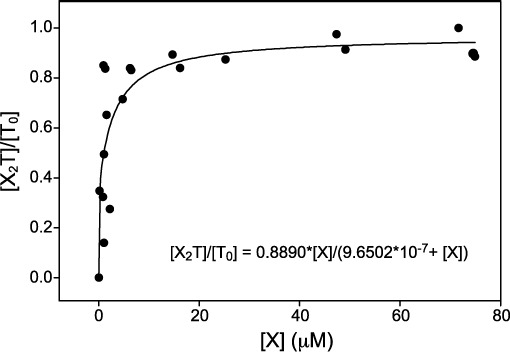
Determination of the binding constant of XR5944 with TFF1-ERE DNA using EtBr FID assay. The concentrations of DNA and EtBr are 16.7 μM and 1 mM, respectively. Binding is presented as the fraction of TFF1-ERE bound versus the concentration of free XR5944. Data were fit to the equation [*X*_2_*T*]/[*T*_0_] = *B*[*X*]/(*K*_D_+[*X*]).

The binding constant of XR5944 was determined by FID assay based on previously established methods using EtBr (34–35). Binding of XR5944 to TFF1-ERE can displace intercalated EtBr and quench the fluorescence from the EtBr–DNA complex, thus allow the measurement of binding fraction. Based on the NMR titration data (Supplementary Figure S1A), the binding constants of XR5944 at the two binding sites appeared to be very similar, as the new peaks from both drug binding sites emerged together. With the approximation of *K*_D1_ = *K*_D2_, the equation [*X*_2_*T*]/[*T*_0_] = *B*[*X*]/(*K*_D_+[*X*]) can be used to determine the *K*_D_ value (see Supplementary Information). Binding fraction data and corresponding free XR5944 concentrations were fit to this equation using SigmaPlot 8.0 software (SPSS Inc.), to obtain a *K*_D_ value of approximately 9.65 × 10^−7^ M (Figure 7). This *K*_D_ value appears to be consistent with the previous data on XR5944 (36).

## DISCUSSION

We have previously shown that XR5944, a DNA bis-intercalator with potent anticancer activity, is capable of inhibiting ERα-mediated transcriptional responses via its ability to block the binding of ERα to the ERE sequence (10). This novel mechanism of action of targeting ERE DNA may be used to overcome the drug resistance of currently available antiestrogen treatments, which all target the hormone-receptor complex (11). Solving the structure of a 2:1 complex of XR5944 with TFF1-ERE DNA by NMR allows us to address two important questions: (i) what are the specific molecular recognition determinants between the two XR5944 molecules and the TFF1-ERE DNA? and (ii) what insights does this structure provide for future structure-based rational drug design of bis-intercalator molecules that target the ERE sequences to modulate ERα-induced transcriptional activity? The structure of the 2:1 complex of XR5944 with TFF1-ERE is the first complex structure of a bis-intercalator with a naturally occurring ERE sequence. The overall structures of the two XR5944 complexes exhibit some important and unexpected differences.

### Both bis-intercalation sites of the two XR5944 molecules with the ERE involve the tri-nucleotide spacer

The consensus ERE is an inverted repeat comprised of two ERE half-sites separated by three bases: AGGTCAnnnTGACCT (18). Historically, the tri-nucleotide ‘nnn’ spacer was considered to be irrelevant to ERα-DNA binding; however, recently we demonstrated that the sequence of the spacer is non-random and modulates receptor-mediated transcriptional response (37–38). Furthermore, we found that the tri-nucleotide spacer plays a clear role in determining the binding affinity of XR5944 to ERE sequences, which is correlated with XR5944 inhibitory effects on ERα-mediated transcriptional activity (21). In particular, we found that XR5944 prefers consensus ERE sequences with the tri-nucleotide spacer sequence CGG for better binding affinity (21). The ERE element of the naturally occurring estrogen-responsive *TFF1* gene contains a 5′ consensus ERE half-site, a non-consensus 3′ half-site and a CGG spacer (Figure 1B). XR5944 binds with high affinity to TFF1-ERE, thus TFF1-ERE appears to be an ideal candidate for drug complex structural determination to understand the specific binding characteristics of XR5944 with EREs. Our NMR solution structure reported in this study showed that two XR5944 molecules bis-intercalate the TFF1-ERE DNA at two adjacent sites (Figure 1B), both involving the tri-nucleotide spacer. It is thus not surprising that the tri-nucleotide spacer sequences can affect the XR5944 binding to EREs.

### Unexpected bis-intercalation sites of XR5944

Previously, we have showed that the preferred bis-intercalating sequence of XR5944 is the 5′-T|GC|A with two symmetric 5′-(TpG):(CpA) binding sites in a 6-mer palindromic DNA (17). The TFF1-ERE sequence contains a 5′-CpA and a 5′-TpG site at each of the half-sites 5′-(AGGTCACGGTGGCCA). However, the preferred bis-intercalating sequence 5′-TGCA of XR5944 is not present in the ERE sequence. It needs to be noted that while the 5′-CpA and 5′-TpG sites are equivalent for a single intercalating site, they are not equivalent in a bis-intercalation binding, which is directional due to the presence of the linker. For example, the different arrangements of a 5′-CpA and a 5′-TpG would result in completely different bis-intercalation binding of XR5944. At 5′-TGCA, the two intercalating phenazine moieties wrap around two central G:C base pairs, whereas at 5′-CATG, the two intercalating phenazine moieties wrap around two central A:T base pairs. Indeed, our results showed that the binding characteristics of XR5944 to the TFF1-ERE sequence were different from its preferred bis-intercalating sequence 5′-TGCA. Specifically, in each drug–DNA complex within the TFF1-ERE DNA, XR5944 binds strongly at one intercalation site but weakly at the second intercalation site (Figure 1B). For the first XR5944 molecule XR1's binding site, 5'-C_5_A_6_C_7_G_8_, the 5′-C_7_pG_8_ appeared to be the strong intercalation site for XR1–2; the 5′-C_5_pA_6_, which is not equivalent to 5′-TpG due to the linker direction in a bis-intercalator, appeared to be the weak intercalation site for XR1–1. It is noteworthy that the 5′-CpG has been indicated to favor XR5944 binding (36).

Surprisingly, the bis-intercalation site of the second XR5944 molecule (XR2) is 5′-G_9_T_10_G_11_G_12_, with the XR2–1 binding site 5′-G_9_pT_10_ being the strong binding site (Figure 1B). The G_9_pT_10_ and G_11_pG_12_ sites are both purine-N steps that have been shown not to favor intercalation binding as they are less flexible in accommodating the structure distortion induced by drug intercalation (39–42). It was thus surprising that the second XR5944 did not bind at the 5′-T_10_G_11_G_12_C_13_ site, which is one base downstream but contains a preferred intercalation site of 5′-TpG (Figure 1B). One explanation is that the second binding site is dependent on the position of the first. In this case, 5′-C_5_pA_6_C_7_pG_8_ is likely to be the more stable and first occupied binding site of XR5944, given its favorable intercalation interactions. Binding of XR5944 at this first binding site results in a negative supercoiling (unwinding) of DNA, which propagates to the adjacent steps, but disappears or even becomes positive supercoiling at a more distant site, thus disfavoring the second drug binding at the farther site 5′-T_10_G_11_G_12_C_13_. The DNA unwinding induced by XR5944 has been observed in a topoisomerase I DNA unwinding/intercalating assay, using negatively supercoiled pBR322 DNA (43). It is noteworthy that the sequence of the first binding site in TFF1-ERE is the consensus ERE half-site (Figure 1B), it is thus expected that XR5944 binds at the same sites in the consensus ERE with a CGG spacer. Indeed, this was supported by our NMR titration data, which showed very similar spectral patterns between the consensus ERE with a CGG spacer and the TFF1-ERE at 2 equivalence of XR5944 (Supplementary Figure S1B).

### Diverse intercalation modes of the XR5944 phenazine rings

In the present complex structures with TFF1-ERE DNA, both XR5944 molecules bind in a parallel base-stacking intercalation mode at the strong binding sites (Figure 6B and C). However, in contrast to the previously observed symmetric bis-intercalation of XR5944 at the preferred 5′-TGCA sequence (17), the binding positions of the XR5944 phenazine rings appear to be diverse and are not always symmetric within the intercalation sites (Figure 6D and E). For the first strong binding site (C_7_pG_8_):(C_23_pG_24_), the XR1–2 phenazine chromophore stacks more extensively within the C_23_pG_24_ step than the C_7_pG_8_ step and is positioned clearly closer to the C_23_pG_24_ sugar backbone (Figure 6B, D and F). At the second strong binding site, (G_9_pT_10_):(A_21_pC_22_), a more symmetric intercalation was observed for the XR2–1 (Figure 6C, E and G). While the intercalation conformations of the two weak binding sites were not as well defined, XR1–1 appears to more extensively intercalate within the (C_5_pA_6_):(T_25_pG_26_) step as compared to XR2–2 at the (G_11_pG_12_):(C_19_pC_20_) binding site (Figure 6F and G). More remarkably, the intercalating phenazines adopt different orientations in the three drug complexes, i.e. XR1, XR2 and XR5944 with preferred 5′-TGCA sequence. In the XR5944 complex with the ideal 5′-TGCA binding site (17), the XR5944 phenazine intercalates with its ring A (where the linker connects) close to the descending DNA strand viewing into the major groove, therefore, the linker connecting the two phenazine rings adopts a left-handed twist (Supplementary Figure S4C). In contrast, in the XR1 complex with the TFF1-ERE sequence, the two phenazine rings adopt an opposite intercalation orientation, with the ring A close to the opposite DNA strand (ascending), thus resulting in a different linker conformation of a right-handed twist (Figure 6B and Supplementary Figure S4A). For the XR2 complex, although the tight binding phenazine XR2–1 intercalates with a similar orientation to that of XR5944 at the ideal binding site 5′-TGCA, the poor phenazine intercalation at the weak binding site of XR2–2 results in a linker conformation running more vertically across the major groove (Figure 6C and Supplementary Figure S4B). Therefore, it appears that the different intercalation orientations of the XR5944 phenazine rings determine the linker conformation in the major groove.

### Major-groove DNA-linker interactions of XR5944

Two H-bond acceptors are present in the DNA major groove, i.e. thymine O4 and guanine O6, which can form H-bonds with the linker γ-amino groups of XR5944. In the ideal binding site 5′-TGCA, the two XR5944 phenazine rings wrap around the two central G:C base pairs, whose H-bond acceptors guanine O6 are symmetrically located on the different DNA strands (Supplementary Figure S4D); guanine O6 has been known to be better accessible for major groove H-bonding interactions. In contrast, in both XR1 and XR2 binding sites within the TFF1-ERE DNA, the major groove H-bond acceptors are located on the same DNA strand, i.e. G_24_O6 and T_25_O4 of the XR1 binding site and T_10_O4 and G_11_O6 of the XR2 binding site (Figure 1B), which are not as accessible for the major groove H-bonding interactions. The location of the major groove H-bond acceptors at the non-ideal XR1 and XR2 binding sites appears to determine the asymmetric positioning of the phenazine moieties at the intercalation sites. For example, in the XR1 binding site (C_5_|pA_6_C_7_|pG_8_):(C_23_|pG_24_T_25_|pG_26_) (Figure 6B), both XR1–1 and XR1–2 phenazines are closer to the C_23_|pG_24_T_25_|pG_26_ strand (Figure 6D), to facilitate H-bond interactions with G_24_O6 and T_25_O4. The location of the major groove H-bond acceptors at the non-ideal XR1 and XR2 binding sites may also determine the different phenazine orientations at the intercalation sites (see the last section). For example, in the XR2 binding site (G_9_|pT_10_G_11_|pG_12_):(C_19_|pC_20_A_21_|pC_22_), the two H-bond acceptors T_10_O4 and G_11_O6 are located on the opposite strand from the XR1 binding site, which may result in the different phenazine orientation of XR2 to facilitate better access of its linker to the major groove H-bond acceptors (Figure 6B, 6C).

It is noted that, while the O6 atoms of the two adjacent guanines on the complementary strands provide the most favorable major groove hydrogen bonding interactions (17), the O6 atoms of the two adjacent guanines on the same DNA strand are much less accessible, so a bis-intercalation sandwiching a central GpG sequence would be disfavored. Instead, a thymine O4 and guanine O6 on the same DNA strand may be better accessible to the linker H-bonding interactions, as observed in the XR1 and XR2 complexes. This may be another reason to disfavor the binding of XR2 at the 5′-T_10_G_11_G_12_C_13_ site, which contains a central GpG sequence (Figure 1B). Thus it appears that the combined linker major groove interactions and phenazine intercalation interactions determine the binding sites of XR5944 at a non-ideal binding sequence; to accommodate better major groove hydrogen bonding interactions of the linker, the phenazine moiety may deviate from an ideal intercalation interaction, resulting in different orientations and asymmetric positioning, as well as weak and strong binding sites.

### Design of better bis-intercalator compounds targeting ERE sequence

Understanding the binding mode of XR5944 to a naturally occurring ERE sequence could provide a useful basis for the design of XR5944 derivatives targeting ERE. Our results suggest that the improved binding specificity of XR5944-derived DNA bis-intercalators with the ERE sequence may be obtained through optimization of aminoalkyl linker and intercalation at the weak binding sites. Amine hydrogen bonding appears to be a stabilizing factor for both binding sites. However, XR1 and XR2 show differing hydrogen bonding to their respective sequences, which may be optimized by linker modifications. Flexible linkers have long been known to improve binding affinity at the cost of sequence selectivity (44), so greater sequence recognition may be achieved through utilization of a rigid linker, as previously utilized by several DNA intercalators (45–47). In addition, the length of the aminoalkyl chain may be modified to optimize hydrogen bonding interactions to a specific targeted sequence such as ERE. Moreover, neither XR1–1 nor XR2–2 exhibits strong intercalation interactions at their respective binding site, which may be improved by substitution of the weakly binding phenazine moiety with other known intercalating moieties to achieve a stronger intercalation interaction.

The two XR5944 complex structures within the TFF1-ERE sequence indicated that the binding site and mode of XR5944 within a native DNA promoter sequence is context- and sequence-dependent, and can be different from its ideal binding site shown in artificially designed short palindromic sequences. In addition, a drug bis-intercalation at one site can influence the binding of the second drug at an adjacent intercalation site. Furthermore, it is important to note that a bis-intercalation site is directional with properties that are different from the simple addition of the two single intercalating moieties. These properties could be important considerations for designing of ERE-specific DNA bis-intercalators.

## ACCESSION NUMBER

PDB ID: 2mg8.

## SUPPLEMENTARY DATA

Supplementary Data are available at NAR Online, including [1, 2].

SUPPLEMENTARY DATA
