# Supplementary material for: Solution structure of a 2:1 complex of anticancer drug XR5944 with TFF1 estrogen response element: insights into DNA recognition by a bis-intercalator
Source: Nucleic Acids Res. 2014 Apr 7;42(9):6012–24. doi: 10.1093/nar/gku219 (PMC4027214; doi:10.1093/nar/gku219)
Supplement: SUPPLEMENTARY DATA [file supp_42_9_6012__index.html]

Solution structure of a 2:1 complex of anticancer drug XR5944 with TFF1 estrogen response element: insights into DNA recognition by a bis-intercalator — SUPPLEMENTARY DATA 

# Solution structure of a 2:1 complex of anticancer drug XR5944 with TFF1 estrogen response element: insights into DNA recognition by a bis-intercalator

## SUPPLEMENTARY DATA

**Files in this Data Supplement:**

- SUPPLEMENTARY DATA
