# Supplementary material for: Solution structure of a 2:1 complex of anticancer drug XR5944 with TFF1 estrogen response element: insights into DNA recognition by a bis-intercalator
Source: Nucleic Acids Res. 2014 Apr 7;42(9):6012–24. doi: 10.1093/nar/gku219 (PMC4027214; doi:10.1093/nar/gku219)
Supplement: SUPPLEMENTARY DATA [file supp_gku219_nar_03608_f_2013_File010.pdf]

Supplementary Information

Molecular Recognition of TFF1 Estrogen Response Element by a DNA Bis-intercalating Anticancer Drug XR5944

Clement Lin [a], Raveendra I. Mathad [a], Zhenjiang Zhang [a], Neil Sidell [b], Danzhou Yang [a,c,d,e,] *

METHODS

Binding constant determination by fluorescent intercalator displacement (FID) assay.

The binding constant of XR5944 was determined by FID assay based on previously established methods using ethidium bromide (EtBr) (1,2). XR5944 binding to TFF1-ERE would displace intercalated EtBr and quench the fluorescence from the EtBr-TFF1 complex and allow the measurement of binding fraction. The binding constant was determined based on the following binding model, where X and T represent XR5944, and TFF1-ERE respectively.

$$2X + T \leftrightarrow X + XT \leftrightarrow X_2T \qquad (1)$$

Based on this, we derive the following:

$$K_{D1} = [X][T]/[XT] \qquad (2)$$

$$K_{D2} = [X][XT]/[X_2T] \qquad (3)$$

The bound fraction at each site, F_1 and F_2 can be defined by the following equations:

$$F_1 = [XT]/([T]+(XT)) \qquad (4)$$

$$F_2 = [X_2T]/([XT]+(X_2T)) \qquad (5)$$

The binding fractions can be related to K_{D1} and K_{D2} with the following relationships derived from Eqs. 2 and 3.

$$[XT] = [X][T]/K_{D1} \qquad (6)$$

$$[X_2T] = [X][XT]/K_{D2} \qquad (7)$$

Substituting Eqs. 6 and 7 into 4 and 5 and simplifying yields the following relationships:

$$F_1 = [X]/(K_{D1} + [X]) \qquad (8)$$

$$F_2 = [X]/(K_{D2} + [X]) \qquad (9)$$

We can assume that the experimentally determined total bound fraction $[X_2T]/[T_0]$ is the sum of the binding contributions from both binding sites F_1 and F_2. B1 and B2 represent the respective maximum binding contributions.

$[X_2T]/[T_0] = B_1F_1 + B_2F_2 = B_1[X]/(K_{D1}+[X]) + B_2[X]/(K_{D2}+[X])$ (10)

Based on NMR titration data (Figure S1A), XR5944 binds the two binding sites with very similar binding constants. With the approximation of $K_{D1} = K_{D2}$, equation 10 is simplified to the following equation:

$[X_2T]/[T_0] = B[X]/(K_D+[X])$ (11)

Binding fraction data and corresponding free XR5944 concentrations were fit to equation 11 to obtain the K_D using SigmaPlot 8.0 software.

References:

1. Sau, S.P., Kumar, P., Sharma, P.K. and Hrdlicka, P.J. (2012) *Fluorescent intercalator displacement replacement (FIDR) assay: determination of relative thermodynamic and kinetic parameters in triplex formation; a case study using triplex-forming LNAs*. Nucleic Acids Res, **40,** e162.

2. Boger, D.L., Fink, B.E., Brunette, S.R., Tse, W.C. and Hedrick, M.P. (2001) *A simple, high-resolution method for establishing DNA binding affinity and sequence selectivity*. J Am Chem Soc., **123,** 5878-5891.

Table S1. ^{1}H NMR Chemical Shifts (ppm) of the 2:1 XR5944-TFF1 Complex and the Free TFF1 DNA (in parentheses) at 25 ^{o}C

	H1'	H2'	H2''	H3'	H4'	H5', H5''	H2 / H5 / HMe	H6 / H8	H1 / H3
**A1**	5.85	2.28	2.47	4.71	4.26	3.59, 4.08	7.83	7.87	
	(5.85)	*(2.27)*	*(2.46)*	*(4.70)*	*(4.25)*	*(3.95, 4.08)*	*(7.87)*	*(7.87)*	
**G2**	5.48	2.61	2.64	4.87	4.26	4.00, 4.09		7.73	12.52
	(5.47)	*(2.61)*	*(2.65)*	*(4.86)*	*(4.26)*	*(3.98, 4.08)*		*(7.74)*	*(12.69)*
**G3**	5.90	2.45	2.67	4.79	4.32	4.04, 4.11		7.58	12.45
	(5.91)	*(2.45)*	*(2.68)*	*(4.79)*	*(4.33)*	*(3.96, 4.09)*		*(7.59)*	*(12.75)*
**T4**	5.93	2.01	2.39	4.76	4.13	4.04, 4.05	1.16	7.15	13.35
	(5.96)	*(2.01)*	*(2.41)*	*(4.78)*	*(4.14)*	*(4.05, 4.13)*	*(1.19)*	*(7.16)*	*(13.46)*
**C5**	5.41	1.88	2.23	4.74	4.31	3.98, 4.04	5.49	7.36	
	(5.42)	*(1.95)*	*(2.27)*	*(4.75)*	*(4.14)*	*(3.99, 4.03)*	*(5.56)*	*(7.40)*	
**A6**	5.93	2.55	2.66	4.90	4.27	3.98, 4.03	8.03	8.08	
	(6.05)	*(2.60)*	*(2.75)*	*(4.83)*	*(4.31)*	*(3.98, 4.03)*	*(7.54)*	*(8.16)*	
**C7**	5.79	2.28	1.89	4.66	4.04	4.00, 4.01	5.11	7.14	
	(5.43)	*(2.15)*	*(1.71)*	*(4.91)*	*(4.00)*	*(4.01, 4.12)*	*(5.13)*	*(7.05)*	
**G8**	5.46	2.36	2.41	4.74	4.10	3.99, 4.02		7.51	10.83
	(5.52)	*(2.55)*	*(2.62)*	*(4.85)*	*(4.21)*	*(3.94)*		*(7.68)*	*(12.78)*
**G9**	5.93	2.03	2.40	4.77	4.14	4.00, 4.02		7.14	10.80
	(5.81)	*(2.37)*	*(2.61)*	*(4.78)*	*(4.27)*	*(3.99, 4.06)*		*(7.52)*	*(12.65)*
**T10**	5.20	2.06	2.28	4.64	4.14	3.96, 4.01	1.61	7.20	10.92
	(5.65)	*(1.82)*	*(2.25)*	*(4.73)*	*(4.02)*	*(4.00, 4.08)*	*(1.22)*	*(6.97)*	*(13.43)*
**G11**	5.51	2.54	2.62	4.87	4.25	4.05, 4.09		7.67	12.18
	(5.52)	*(2.50)*	*(2.57)*	*(4.87)*	*(4.22)*	*(4.03, 4.07)*		*(7.70)*	*(12.72)*
**G12**	5.76	2.46	2.59	4.87	4.29	3.99, 4.10		7.60	12.76
	(5.79)	*(2.46)*	*(2.60)*	*(4.85)*	*(4.30)*	*(3.95, 4.08)*		*(7.62)*	*(12.84)*
**C13**	5.78	1.86	2.26	4.66	4.29	4.02, 4.13	5.20	7.21	
	(5.82)	*(1.88)*	*(2.27)*	*(4.68)*	*(4.30)*	*(4.03)*	*(5.22)*	*(7.24)*	
**C14**	5.67	1.93	2.20	4.67	4.13	3.95, 4.05	5.55	7.36	
	(5.70)	*(1.94)*	*(2.21)*	*(4.69)*	*(4.05)*	*(3.91, 3.97)*	*(5.58)*	*(7.37)*	
**A15**	6.24	2.39	2.59	4.60	4.10	3.97, 4.00	7.74	8.17	
	(6.26)	*(2.39)*	*(2.58)*	*(4.60)*	*(4.09)*	*(3.97, 4.01)*	*(7.74)*	*(8.17)*	
**T16**	5.76	1.67	2.11	4.51	3.94	3.55, 3.56	1.49	7.24	12.75
	(5.76)	*(1.66)*	*(2.11)*	*(4.50)*	*(3.93)*	*(3.91)*	*(1.50)*	*(7.24)*	
**G17**	5.51	2.63	2.69	4.86	4.23	3.86, 3.93		7.84	12.61
	(5.52)	*(2.64)*	*(2.70)*	*(4.86)*	*(4.23)*	*(3.86, 3.93)*		*(7.86)*	*(12.92)*
**G18**	5.77	2.27	2.51	4.87	4.23	3.89, 4.12		7.68	12.71
	(5.80)	*(2.30)*	*(2.58)*	*(4.87)*	*(4.23)*	*(4.08)*		*(7.70)*	*(12.89)*
**C19**	5.78	1.94	2.28	4.86	4.08	3.90, 4.04	5.12	7.22	
	(5.88)	*(1.97)*	*(2.36)*	*(4.88)*	*(4.12)*	*(3.96, 4.03)*	*(5.21)*	*(7.26)*	
**C20**	5.28	1.53	2.05	4.65	3.91	3.89, 3.96	5.31	7.15	
	(5.31)	*(1.94)*	*(2.25)*	*(4.71)*	*(3.95)*	*(3.96, 3.98)*	*(5.50)*	*(7.36)*	
**A21**	5.96	2.77	2.73	5.05	4.24	3.97, 4.04	7.05	8.11	
	(6.12)	*(2.62)*	*(2.79)*	*(4.92)*	*(4.32)*	*(3.97, 4.04)*	*(7.63)*	*(8.16)*	
**C22**	5.65	1.96	2.29	4.53	4.11	3.86, 4.03	5.59	7.35	
	(5.70)	*(1.87)*	*(2.28)*	*(4.66)*	*(4.06)*	*(3.96, 4.03)*	*(5.15)*	*(7.14)*	
**C23**	5.86	2.19	2.46	4.75	4.07	3.98, 4.04	5.15	7.16	
	(5.82)	*(2.48)*	*(2.61)*	*(4.70)*	*(4.04)*	*(3.95, 3.97)*	*(5.37)*	*(7.26)*	
**G24**	5.69	2.38	2.60	4.76	4.29	3.99, 4.09		7.70	10.54
	(5.83)	*(2.48)*	*(2.63)*	*(4.73)*	*(4.24)*	*(3.99, 4.09)*		*(7.75)*	*(12.61)*
**T25**	5.45	1.77	2.18	4.67	4.01	3.97, 4.10	1.14	6.88	11.15
	(5.61)	*(1.78)*	*(2.21)*	*(4.85)*	*(4.10)*	*(4.00, 4.07)*	*(1.33)*	*(6.96)*	*(13.35)*
**G26**	5.39	2.54	2.63	4.88	4.20	3.99, 4.09		7.69	12.05
	(5.39)	*(2.57)*	*(2.64)*	*(4.86)*	*(4.22)*	*(3.92, 4.00)*		*(7.75)*	*(12.44)*
**A27**	6.07	2.55	2.76	4.89	4.33	4.10, 4.19	7.73	8.01	
	(6.10)	*(2.57)*	*(2.76)*	*(4.89)*	*(4.35)*	*(4.02, 4.10)*	*(7.71)*	*(8.04)*	
**C28**	5.70	1.88	2.28	4.65	4.17	4.00, 4.03	5.10	7.12	
	(5.71)	*(1.88)*	*(2.28)*	*(4.67)*	*(4.17)*	*(4.01, 4.04)*	*(5.13)*	*(7.13)*	
**C29**	5.99	2.19	2.38	4.71	4.08	3.96, 4.06	5.54	7.49	
	(6.00)	*(2.19)*	*(2.38)*	*(4.71)*	*(4.07)*	*(4.02, 4.05)*	*(5.56)*	*(7.49)*	
**T30**	6.16	2.19	2.20	4.46	3.99	3.97, 4.02	1.66	7.49	12.03
	(6.16)	*(2.19)*	*(2.21)*	*(4.46)*	*(3.99)*	*(3.97)*	*(1.67)*	*(7.49)*	

	H2	H3	H4	H6	H7	H8	HMe	Hα	Hβ	Hδ
**X1-1**	7.46	7.28	7.8	6.76	6.584	7.05	2.34	3.46	3.24	3.1
**X1-2**	7.51	7.4	7.76	6.71	6.542	6.988	2.29	3.303, 3.857	3.19	3.08
**X2-1**	7.41	7.21	7.68	7.06	6.716	6.73	2.28	3.287, 3.645	3.23	3.09
**X2-2**	7.52	7.33	7.82	6.74	6.577	7.025	2.33	3.47	3.25	3.08

Table S2. Base pair step parameters of TFF1-ERE DNA in the 2:1 XR5944-DNA complex. [a]

**Step ID**	**Step**	**Pair ID1**	**Pair ID2**	**Shift**	**Slide**	**Rise**	**Tilt**	**Roll**	**Twist**
1	AG/CT	1	2	-1.01	0.29	3.71	-0.5	19.11	27.65
2	GG/CC	2	3	0.98	-0.62	3.93	1.6	0.93	34.92
3	GT/AC	3	4	-0.67	-1.2	3.6	-0.54	-10.03	33.27
4	TC/GA	4	5	1.21	0.75	3.7	-0.53	-0.96	30.81
5	CA/TG	5	6	-1.74	1.68	5.76	-2.94	-7.14	23.57
6	AC/GT	6	7	-0.12	-0.16	3.69	0.45	-6.66	29.67
7	CG/CG	7	8	-0.89	-0.73	6.09	-3.43	7.55	24.26
8	GG/CC	8	9	0.03	-0.73	3.2	-0.81	8.26	33.04
9	GT/AC	9	10	-0.85	-0.4	6.13	-1.73	-5.69	22.97
10	TG/CA	10	11	0.92	-0.18	3.67	1.48	0.27	26.77
11	GG/CC	11	12	-0.95	1.62	5.45	-6.43	-8.86	25.88
12	GC/GC	12	13	0.39	-0.33	3.62	0.38	-3.21	39.79
13	CC/GG	13	14	0.37	-0.15	3.72	0.65	1.61	28.17
14	CA/TG	14	15	-0.8	0.22	3.92	-0.03	-6.35	40.81

[a.] Generated by Web 3DNA (Zheng et al., 2009)

Reference:

Zheng, G., Lu, X.J., and Olson, W.K. (2009) *Web 3DNA—a web server for the analysis, reconstruction, and visualization of three-dimensional nucleic-acid structures*. Nucleic Acids Res, **37,** (suppl 2): W240-W246.

Table S3. Individual base pair parameters of TFF1-ERE DNA in the 2:1 XR5944-DNA complex. [a]

**Pair ID**	**Base Pair**	**Shear**	**Stretch**	**Stagger**	**Buckle**	**Propeller**	**Opening**
1	A-T	1.13	-0.01	0	0.15	-0.64	-9.03
2	G-C	-0.21	-0.25	-0.02	-0.52	-0.4	-12.36
3	G-C	-0.05	0.01	0	-0.12	-0.46	-2.28
4	T-A	-0.01	-0.19	0.02	-0.57	-0.2	-8.69
5	C-G	0.85	-0.28	-0.03	1.4	-0.26	-0.06
6	A-T	-0.46	-0.3	0	-0.25	-0.51	-8.23
7	C-G	-0.62	-0.14	-0.02	0.93	-0.92	-2.69
8	G-C	0.18	-0.07	0.05	0.62	0.45	-9.09
9	G-C	0.11	-0.11	0	-0.34	-1.42	-9.42
10	T-A	0.09	0.09	0.06	-0.92	0.14	-5.64
11	G-C	0.62	0.04	0	0.15	-0.44	-2.33
12	G-C	-0.01	-0.33	-0.04	-0.53	0.01	-10.68
13	C-G	0.57	-0.29	-0.01	0.64	-0.82	-3.93
14	C-G	-0.44	-0.08	0.01	-0.17	-0.85	-5.29
15	A-T	0.13	-0.04	-0.02	-0.16	0.16	-13.14

[a.] Generated by Web 3DNA.

FIGURE LEGENDS

Figure S1. Imino proton region of the 1D ^{1}H NMR titration spectra of TFF1-ERE DNA (A) and the consensus ERE sequence with a CGG spacer (B) with XR5944. . Conditions: 25°C, pH7, 50 mM sodium phosphate solution.

Figure S2. The base H8/H6 proton assignments using 1D ^{15}N-edited HMQC experiments on site-specific labeled TFF1 DNA for free TFF1-ERE DNA (A) and 2:1 XR5944:TFF1 complex (B).

Figure S3. The expanded aromatic-H1' region of the 2D-NOESY spectrum of the free TFF1-ERE DNA duplex. The sequential assignment pathways are shown for the sense strand (A_1-A_{15}) (A) and the complement strand (T_{16}-T_{30}) (B). The intra-residue TFF1 DNA H8/H6-H1' NOEs are labeled with residue names.

Figure S4. The major groove view of the binding of the first XR5944 molecule XR1 (A) and the second XR5944 molecule XR2 (B) in the 2:1 XR5944:TFF1 complex, and the binding of XR5944 with its preferred bis-intercalation site 5'-TGCA (C). XR5944 molecules are shown in CPK model. (D) The stereo view of the H-bonding interactions (black dashed lines) between the carboxamide aminoalkyl linker of XR5944 and the DNA major groove at the preferred bis-intercalation site 5'-TGCA. Carbon atoms of XR5944 are colored pink, whereas carbon atoms of DNA are colored green.

Consensus ERE CGG spacer
5'-AGGTCA CGG TGACCT-3'
3'-TCCAGT GCC ACTGGA-5'

TFF1 ERE
5'-AGGTCA CGG TGGCCA-3'
3'-TCCAGT GCC ACCGGT-5'

A. TFF1-ERE

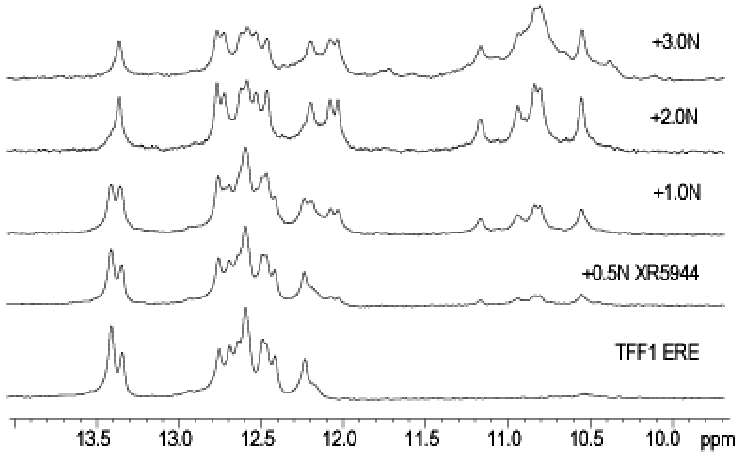

B. Consensus ERE with CGG spacer

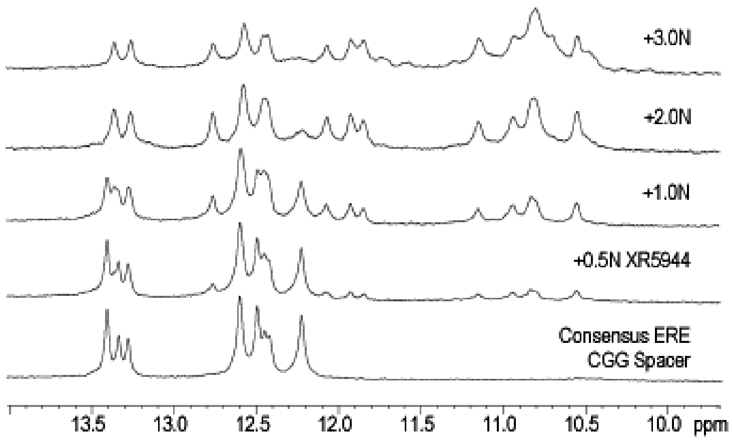

Figure S1.

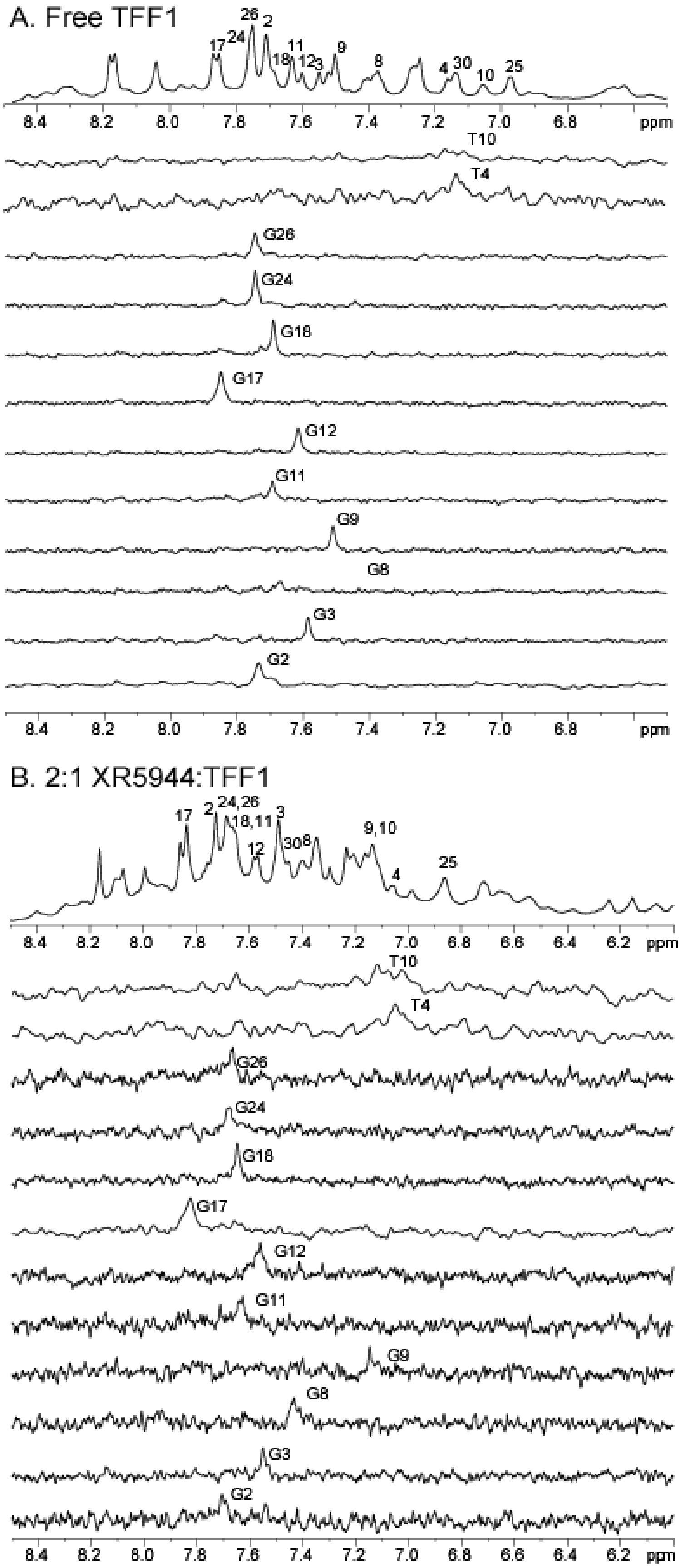
A. Free TFF1
26 2
17 24
11
18
12
3
9
8
4 30
10
25
8.4 8.2 8.0 7.8 7.6 7.4 7.2 7.0 6.8 ppm
T10
T4
G26
G24
G18
G17
G12
G11
G9
G8
G3
G2
8.4 8.2 8.0 7.8 7.6 7.4 7.2 7.0 6.8 ppm
B. 2:1 XR5944:TFF1
17 2 24,26
18,11 3
12
30 8
9,10
4
25
8.4 8.2 8.0 7.8 7.6 7.4 7.2 7.0 6.8 6.6 6.4 6.2 ppm
T10
T4
G26
G24
G18
G17
G12
G11
G9
G8
G3
G2
8.4 8.2 8.0 7.8 7.6 7.4 7.2 7.0 6.8 6.6 6.4 6.2 ppm

Figure S2.

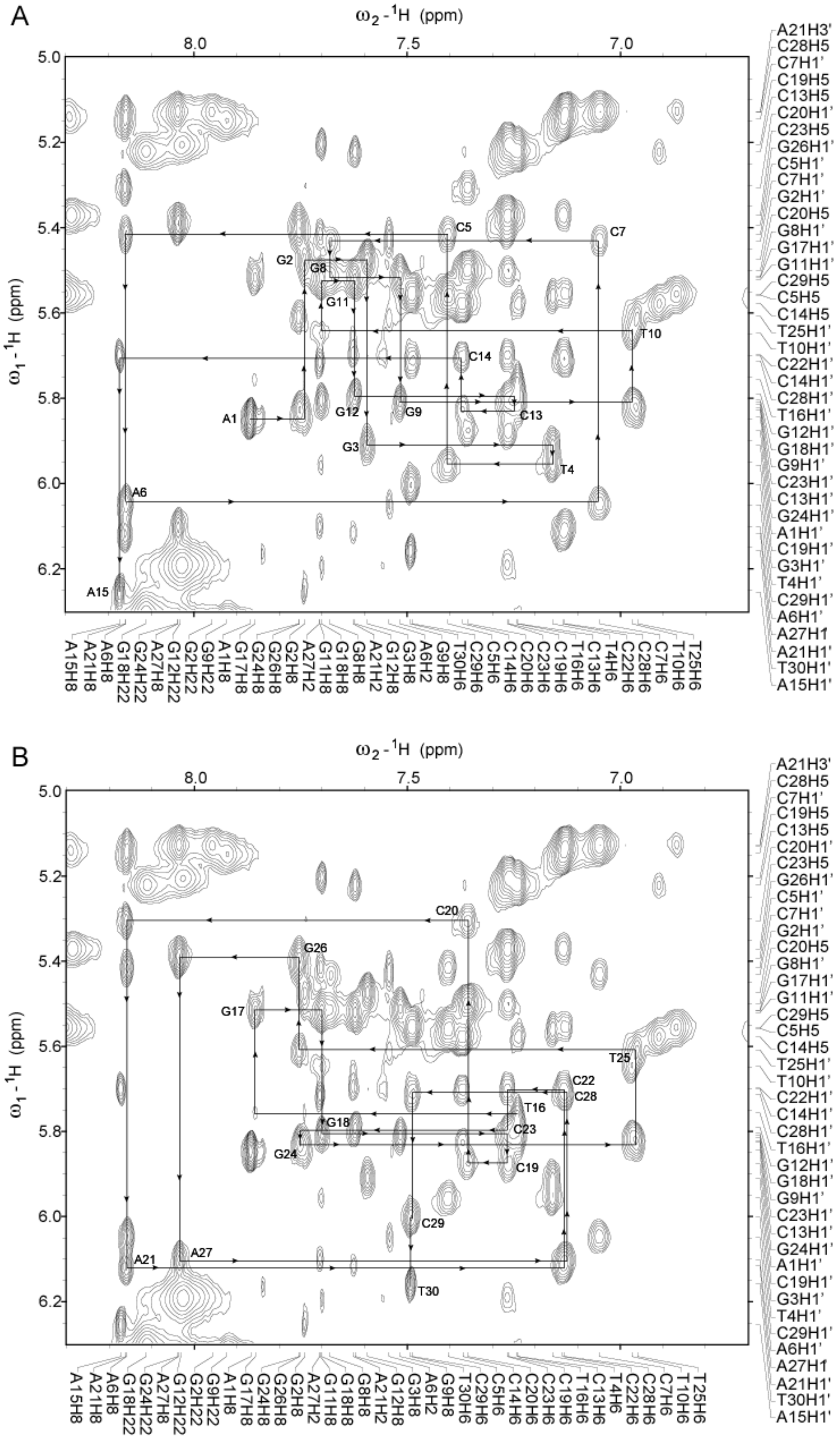
A
B
ω2 - 1H (ppm)
ω1 - 1H (ppm)
8.0
7.5
7.0
5.0
5.2
5.4
5.6
5.8
6.0
6.2
C5
C7
G2
G8
G11
T10
C14
A1
G12
G9
C13
G3
T4
A6
A15
C20
G26
G17
T25
C22
C28
T16
G18
C23
G24
C19
C29
A21
A27
T30
A15H8
A21H8
A6H8
G18H22
G24H22
A27H8
G12H22
G2H22
G9H22
A1H8
G17H8
G24H8
G26H8
G2H8
A27H2
G11H8
G18H8
G8H8
A21H2
G12H8
G3H8
A6H2
G9H8
T30H6
C29H6
C5H6
C14H6
C20H6
C23H6
C19H6
T16H6
C13H6
T4H6
C22H6
C28H6
C7H6
T10H6
T25H6
A21H3'
C28H5
C7H1'
C19H5
C13H5
C20H1'
C23H5
G26H1'
C5H1'
C7H1'
G2H1'
C20H5
G8H1'
G17H1'
G11H1'
C29H5
C5H5
C14H5
T25H1'
T10H1'
C22H1'
C14H1'
C28H1'
T16H1'
G12H1'
G18H1'
G9H1'
C23H1'
C13H1'
G24H1'
A1H1'
C19H1'
G3H1'
T4H1'
C29H1'
A6H1'
A27H1'
A21H1'
T30H1'
A15H1'

Figure S3.

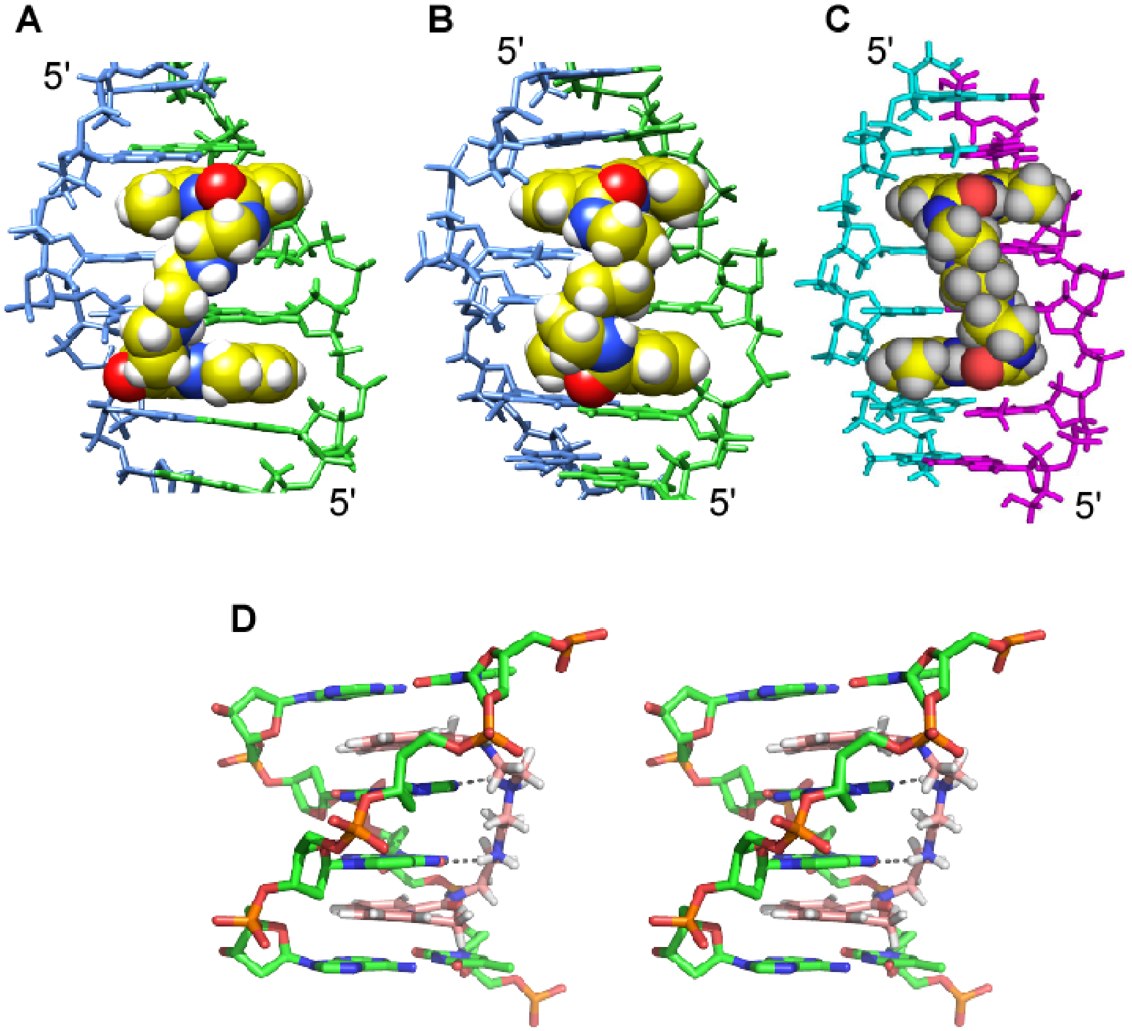

Figure S4.
